# Microbial Ecotoxicology of Marine Plastic Debris: A Review on Colonization and Biodegradation by the “Plastisphere”

**DOI:** 10.3389/fmicb.2019.00865

**Published:** 2019-04-25

**Authors:** Justine Jacquin, Jingguang Cheng, Charlène Odobel, Caroline Pandin, Pascal Conan, Mireille Pujo-Pay, Valérie Barbe, Anne-Leila Meistertzheim, Jean-François Ghiglione

**Affiliations:** ^1^UMR 7621, CNRS, Laboratoire d’Océanographie Microbienne, Observatoire Océanologique de Banyuls-sur-Mer, Sorbonne Université, Banyuls-sur-Mer, France; ^2^Génomique Métabolique, Genoscope, Institut de Biologie François Jacob, Commissariat á I’Énergie Atomique (CEA), CNRS, Université Evry, Université Paris-Saclay, Évry, France; ^3^Plastic@Sea, Observatoire Océanographique de Banyuls-sur-Mer, Banyuls-sur-Mer, France

**Keywords:** bacteria, marine plastics debris, colonization, biodegradation, metabolic pathways

## Abstract

Over the last decades, it has become clear that plastic pollution presents a global societal and environmental challenge given its increasing presence in the oceans. A growing literature has focused on the microbial life growing on the surfaces of these pollutants called the “plastisphere,” but the general concepts of microbial ecotoxicology have only rarely been integrated. Microbial ecotoxicology deals with (i) the impact of pollutants on microbial communities and inversely (ii) how much microbes can influence their biodegradation. The goal of this review is to enlighten the growing literature of the last 15 years on microbial ecotoxicology related to plastic pollution in the oceans. First, we focus on the impact of plastic on marine microbial life and on the various functions it ensures in the ecosystems. In this part, we also discuss the driving factors influencing biofilm development on plastic surfaces and the potential role of plastic debris as vector for dispersal of harmful pathogen species. Second, we give a critical view of the extent to which marine microorganisms can participate in the decomposition of plastic in the oceans and of the relevance of current standard tests for plastic biodegradability at sea. We highlight some examples of metabolic pathways of polymer biodegradation. We conclude with several questions regarding gaps in current knowledge of plastic biodegradation by marine microorganisms and the identification of possible directions for future research.

## Introduction

The amount of land-based plastic debris entering the ocean is estimated at 4.8 to 12.7 million tons per years (Jambeck et al., 2015). It is so important that plastic is regarded as a marker of the Anthropocene (Duis and Coors, 2016; Zalasiewicz et al., 2016). A growing body of research has investigated plastic distribution (Willis et al., 2017; Worm et al., 2017) and toxicity for marine fauna (Bakir et al., 2014; Gewert et al., 2015). A comparatively smaller but growing literature has been devoted to the microbial ecotoxicology of marine plastic debris, i.e. (1) the impact of plastic on marine microbial life together with the various ecosystem services that marine microbial life ensures and inversely, (2) the role of microorganisms in the degradation of ocean plastic (Ghiglione et al., 2014, 2016). Both aspects will be successively explored by this review, which covers the last 15 years of literature.

The investigation of microorganisms colonizing plastic surfaces using modern techniques of massive DNA sequencing (Zettler et al., 2013) was introduced only recently. The authors introduced the world “plastisphere” to describe the microbial life growing on these surfaces. They also detected members of the potentially pathogenic genus *Vibrio*, which may be dispersed over long distances by floating persistent plastics. Since then, several studies investigated various marine environments, such as the North Pacific Gyre (Debroas et al., 2017) or the Mediterranean Sea (Dussud et al., 2018a). In parallel, a growing literature described the first steps of colonization of new plastic until the formation of a mature biofilm (Lobelle and Cunliffe, 2011; Oberbeckmann et al., 2015; Dussud et al., 2018a).

Such knowledge is of great interest to better understand the impact of plastic on marine microbial life and ecosystem functions. Only one study so far used shotgun metagenomics, showing that plastic-inhabiting microbes present an enriched gene repertoire compared to microbes living in the surrounding waters (Bryant et al., 2016). In this review, we argue that current knowledge is insufficient to draw a clear picture of the impact of plastic on marine microbial life and ecosystem functions, and we propose several directions for further studies in this field (see section “Microorganisms Colonizing Plastic at Sea”).

The role of microbes on plastic degradation in the ocean is a second subject of concern. Very recently, an excellent comprehensive review concluded that “current international standards and regional test methods are insufficient in their ability to realistically predict the biodegradability of carrier bags in marine environment, due to several shortcomings in experimental procedures and a paucity of information in the scientific literature” (Harrison et al., 2018). The capability of microorganisms to biodegrade plastic was reported for numerous bacterial strains (Krueger et al., 2015). Fungi also have the capability to biodegrade plastics, but most of the studies were conducted in terrestrial conditions (Cosgrove et al., 2007; Koitabashi et al., 2012; Gajendiran et al., 2016; Magnin et al., 2018) whereas very few studies so far exist in marine conditions (Gonda et al., 2000; Pramila and Ramesh, 2011). Moreover, most of these studies were based on the selection and testing of single strains in laboratory conditions, which is very far from environmental conditions. In this review, we underscore the knowledge gaps on plastic biodegradation by marine microorganisms and we attempt to identify possible directions for future research in this area (see section “How Much Can Microorganisms Participate in Plastic Degradation at Sea?”).

## Microorganisms Colonizing Plastic at Sea

### A New Niche for Marine Microorganisms

It was not until recently that the first work using modern techniques of massive DNA sequencing provided a detailed picture of the microbial life on plastic and introduced the term “plastisphere” (Zettler et al., 2013). Bacteria, Archaea, Fungi and microbial Eukaryotes were detected in several studies, starting from plastics sampled at sea or from new plastics experimentally incubated in marine conditions (Table 1). Plastic debris are mainly composed of polyethylene (PE) at sea surface, followed by polypropylene (PP) and polystyrene (PS) (Auta et al., 2017). Whatever the polymer type, recent studies emphasized the difference between the bacteria living on plastics and the bacteria living in free-living state (Debroas et al., 2017) or on organic particles in the surrounding seawater (Dussud et al., 2018a; Oberbeckmann et al., 2018). Similar observations have been made for fungal communities (Kettner et al., 2017).

**Table 1:** List of recent studies using molecular techniques to evaluate the biodiversity of the plastisphere in different geographic regions, for plastic samples taken at sea or incubated in seawater conditions for the purpose of the studies.

Studied area	Sample type	Method	Gene target	Target	References
North Pacific subtropical Gyre	Sampling at sea surface	Metagenomic sequencing		Bacteria and Eukaryote	Bryant et al., 2016
Baltic Sea	Incubation in seawater	V4 18S rRNA sequencing	565-981	Microbial Eukaryote, Fungi	Kettner et al., 2017
Estuary, Baltic Sea	Incubation in seawater	V4 16S rRNA sequencing	515-806	Bacteria and Archaea	Oberbeckmann et al., 2018
North Sea	Incubation in seawater	V4 16S rRNA sequencing	515-806	Bacteria and Archaea	Oberbeckmann et al., 2016
		V9 18S rRNA sequencing	1391-1795	Microbial Eukaryote, Fungi	
North Sea	Sampling at sea surface- Incubation in seawater	DGGE 16S rRNA and sequencing	341-534	Bacteria and Archaea	Oberbeckmann et al., 2014
North Sea	Incubation in seawater and sediment	V3-V4 16S rRNA sequencing	341-785	Bacteria and Archaea	De Tender et al., 2017
		rDNA-ITS2 sequencing		Fungi	
North Atlantic subtropical gyre	Sampling at sea surface	V4 16S rRNA sequencing	515-806	Bacteria and Archaea	Debroas et al., 2017
		V7 18S rRNA sequencing	960-1438	Eukaryote	
North Atlantic	Sampling at sea surface	V4-V6 16S rRNA sequencing	518-1046	Bacteria	Zettler et al., 2013
		V9 16S rRNA sequencing	1380-1510	Microbial Eukaryote	
Mediterranean Sea	Sampling at sea surface	V3-V5 16S rRNA sequencing	515-926	Bacteria and Archaea	Dussud et al., 2018a
Mediterranean Sea	Incubation in seawater	V3-V5 16S rRNA sequencing	515-926	Bacteria and Archaea	Dussud et al., 2018b
Mediterranean Sea	Incubation in seawater	V3-V5 16S rRNA sequencing	515-926	Bacteria and Archaea	Briand et al., 2012
Arabian Sea	Incubation in seawater	V4 16S rRNA sequencing	ND	Bacteria	Muthukrishnan et al., 2018
Estuary, North Sea	Incubation in marine sediment	16S rRNA cloning and sequencing	27-1492	Bacteria	Harrison et al., 2014
Estuary, East China Sea	Sampling at sediment surface	V3-V4 16S rRNA sequencing	319-806	Bacteria	Jiang et al., 2018

*The PCR-amplified regions and the corresponding targeted organisms are indicated. ND, Non-described in the publication.*

Another aspect that received much less attention is the plastisphere living in the water column other than the surface layer. Because of methodological constraints, most of the studies so far have been limited to sampling surface seawater using manta trawls, which represents less than 1% of the global load of plastic in the open ocean (Cózar et al., 2014). Only certain types of plastics made of PE and PP with high surface-to-volume ratios, such as rigid plastics and bundled fishing nets and ropes, have the capability to remain for a very long time at the surface of the oceans (Lebreton et al., 2018). Most other buoyant plastic such as films or smaller pieces, tend to sink to the sediment owing to biofouling (Fazey and Ryan, 2016; Kalogerakis et al., 2017). Very limited information is available concerning the composition of microbial communities on plastic items sampled from the seafloor (De Tender et al., 2015). If photoautotrophic bacteria such as the cyanobacteria of the genera *Phormidium* and *Rivularia* dominate the sub-surface plastisphere communities (Zettler et al., 2013; Bryant et al., 2016; Dussud et al., 2018a), the core microbiome of the seafloor and sub-surface plastisphere seems to share some taxa: Bacteroidetes (*Flavobacteriaceae*) and Proteobacteria (*Rhodobacteraceae* and *Alcanivoracaceae*) (Zettler et al., 2013; Bryant et al., 2016; De Tender et al., 2017; Dussud et al., 2018a).

### Successive Colonization Stages of New Plastics Incubated in Marine Conditions

In parallel to studies on plastic directly sampled at sea, other studies focused on the successive colonization steps of new plastics incubated in marine conditions (Table 1). At sea, plastics are rapidly covered by the “conditioning film” made of inorganic and organic matter, which is then rapidly colonized by bacteria (mainly *Gammaproteobacteria* and *Alphaproteobacteria*) (Oberbeckmann et al., 2015). With time, members of Bacteroidetes become increasingly abundant (Lee et al., 2008). Hydrophobicity and other substratum properties (crystallinity and crystal structure, roughness, glass transition temperature, melting temperature, modulus of elasticity) may play a role in the selection of bacterial community in the early stages of colonization (Pompilio et al., 2008), but probably in a lesser extent when the biofilm becomes mature (Dussud et al., 2018a). The successive growing and maturation phases of biofilm formation, already described for other surfaces such as glass, acryl, steel or rocks and algae (Salta et al., 2013), were also observed for plastics of different compositions (Oberbeckmann et al., 2015). Biofilm developments were followed during several weeks in seawater on PE-based plastic bags (Lobelle and Cunliffe, 2011), polyethylene terephthalate (PET)-based plastic bottles (Oberbeckmann et al., 2014), polyvinyl chloride (PVC) (Dang et al., 2008), or polystyrene (PS) coupons (Briand et al., 2012). PE-based plastics were also rapidly colonized by microorganisms in marine sediments (Harrison et al., 2014). Clear differences in bacterial abundance, diversity and activity were found between non-biodegradable and biodegradable plastics (Eich et al., 2015; Dussud et al., 2018b). Higher colonization by active and specific bacteria were found after six weeks on poly(3-hydroxybutyrate-co-3-hydroxyvalerate) (PHBV) and pre-oxidized PE-based oxodegradable polymers (OXO) in comparison to non-biodegradable PE polymers (Eich et al., 2015; Dussud et al., 2018b). Longer-term studies carried out over a 6-month to one year period also showed differences in biofilm formation and maturation according to the polymer type, i.e. PE, PP, PET, or polycarbonate (PC) (Webb et al., 2009; De Tender et al., 2017). Not only bacteria but also fungi were shown to form biofilms on plastic surfaces (Pramila and Ramesh, 2011), mainly dominated by Chytridiomycota, Cryptomycota (Kettner et al., 2017) and Ascomycota (Oberbeckmann et al., 2016; De Tender et al., 2017; Kettner et al., 2017).

### Potential Impact of Plastic on the Microbial Role in Regulation of Biogeochemical Cycles

**FIGURE 1:**
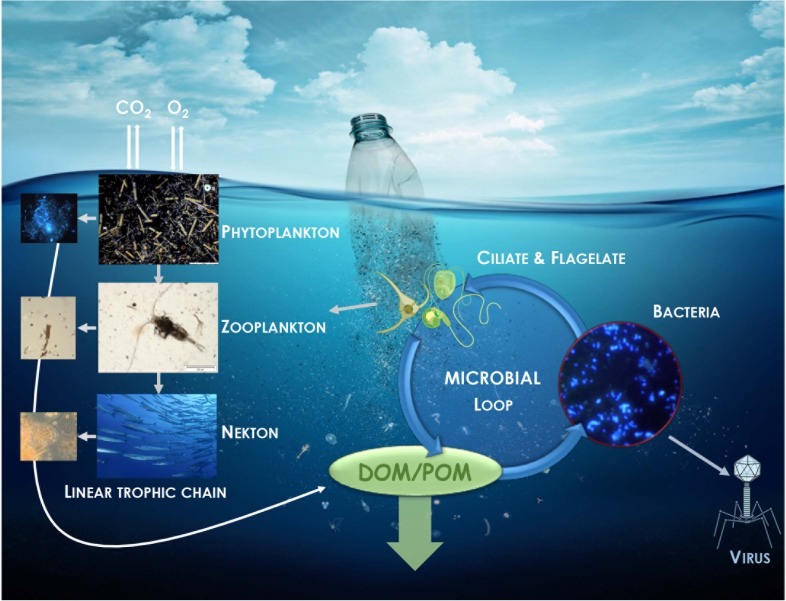
Illustration of the potential impact of plastic in the regulatory role of carbon and nutrient cycles played by bacteria *via* the microbial loop. Dissolve (DOM) and particulate (POM) organic matter originated from the linear trophic chain is returned to higher trophic levels *via* its incorporation in bacterial biomass.

The quantity of plastic in the oceans can no longer be considered as a limited ecological problem, since small pieces of plastic called “microplastics” (<5 mm) found at sea could cover 4.2 million km^2^ of the sea surface (Charette and Smith, 2010; Hidalgo-Ruz et al., 2012; Eriksen et al., 2014). Marine microorganisms that compose the plastisphere are known to play a key role in the biogeochemical cycles in the oceans (Pomeroy et al., 2007). One-half of oceanic primary production on average is channeled *via* heterotrophic bacterioplankton into the microbial loop, thus contributing significantly to food web structure and carbon biogeochemical cycling in the ocean (Fenchel, 2008; Figure 1). Only one recent study compared the heterotrophic production of bacteria living on plastic and in seawater. Heterotrophic bacteria living on plastics were particularly active, the cell-specific activity measured by ^3^H-leucine incorporation into proteins being 43- to 88-fold higher than that of the free-living fraction (Dussud et al., 2018a). Unfortunately, these results were obtained in the frame of a study on colonization of new plastics incubated at sea for a relatively short period (45 days). Similar methodologies applied to plastics that had spent several years at sea would be necessary to evaluate how much the large amount of plastic and the accompanying plastisphere influence the biogeochemical carbon cycle in the oceans.

Interestingly, most of the studies aiming to characterize the plastisphere mentioned that Cyanobacteria were overrepresented on plastics compared to the surrounding free-living and organic particle-attached fractions. The relative importance of photosynthetic activities that Cyanobacteria living on plastic have on global pelagic primary production is still unknown.

Coupling primary production and heterotrophic production measurements over large temporal and spatial scales will be necessary to obtain a better view of the role of the plastisphere on carbon cycling in the oceans. Microorganisms are not only involved in the carbon cycle, but basically in all other biogeochemical cycles including nitrogen, sulfur, iron, manganese, chromium, phosphorus, calcium and silicate cycles, which may also be impacted by the presence of plastic at sea (Hutchins and Fu, 2017).

### Potential Dispersion of Pathogen Species

Interest has been raised about opportunist pathogen dispersal on plastics, such as animal or human pathogenic *Vibrio* sp. (Zettler et al., 2013). Marine plastic debris as vector of harmful species was first suggested by Masó et al. (2003), who identified potential harmful dinoflagellates such as *Ostreopsis* sp. and *Coolia* sp. Putative pathogens of fish (*Tenacibaculum* sp.) and of invertebrates (*Phormidium* sp. and *Leptolyngbya* sp.) were found to be more common on plastic compared to surrounding seawater (Dussud et al., 2018a). Some bacterial taxa considered as putative pathogens for human, coral and fish were also found in the intertidal zone of the Yangtze Estuary, at relatively low abundance (<1.6%) (Jiang et al., 2018). A putative pathogen for coral *Halofolliculina* spp. was found to be abundant on some western Pacific plastic debris (Goldstein et al., 2014). Some toxic eukaryotic species were also mentioned by Debroas et al. (2017) at low abundance (<0.04%), but might be regarded as hitchhiker organisms. Nevertheless, caution should be taken since the 16S rRNA metabarcoding approach used in all these studies was not an appropriate method for describing bacterial virulence. The recent coupling of the 16S rRNA metabarcoding technique with the detection of virulence-associated genes may be an interesting option to address this question (Kirstein et al., 2016). Pathogenicity evidence on marine animals in relation to the plastisphere has never been proven, and further research will be required before publicizing alarmist conclusions on the possible responsibility of plastic debris as vector for the spread of disease-causing organisms. Apart from those results, microplastics colonized by pathogens may also pose threats to humans who are exposed to contaminated beach and bathing environments (Keswani et al., 2016). Evidence is still missing to determine whether plastic debris could lead to the spread and prolonged persistence of pathogenic species in the oceans.

### Factors Driving the Plastisphere Composition and Activities

Factors driving the plastisphere composition are complex, mainly spatial and seasonal, but are also influenced by the polymer type, surface properties and size. Plastisphere communities studied in different polymer types floating in the North Pacific and North Atlantic reflected first their biogeographic origins, and to a lesser extent the plastic type (Amaral-Zettler et al., 2015). Similar conclusions were found for bacterial communities colonizing plastics along an environmental gradient. These communities are shaped firstly by the freshwater to marine environmental conditions and secondarily by the plastic type (PS and PE) (Oberbeckmann et al., 2018). Inversely, another study based on a large number of microplastics sampled in the western Mediterranean Sea showed no effect of geographical location (including coastal and open ocean samples) or plastic type (mainly PE, PP, and PS) on the bacterial community composition. The growing number of studies on the plastisphere are giving a better view of the microbial biofilm community on plastics in the oceans, but the complex network of influences is still the subject of ongoing debate. A clearer picture will hopefully emerge from more extensive investigations with widespread and numerous samples, together with better descriptions of the physical and chemical properties of the polymers.

The physical properties of plastic offer a unique habitat that contribute to the long-distance transport of diverse microbial hitchhikers attached to its surface (Harrison et al., 2011; Zettler et al., 2013). A vast range of other phyla, including Arthropoda, Annelida, Mollusca, Bryozoa, and Cnidaria have conferred on plastics the role of vector for the transfer of organisms, some of them being cataloged as invasive alien species (Oberbeckmann et al., 2015). For instance, plastic debris with tropical biota including corals was detected in the Netherlands (Hoeksema, 2012), and Southern Ocean bryozoans were observed in Antarctica (Barnes and Fraser, 2003). Interactions between micro- and macro-organisms, their substratum and their surroundings are needed to better predict the ecological consequences of microplastics transported through the global oceans.

## How Much Can Microorganisms Participate in Plastic Degradation at Sea?

### Definition and Main Processes Involved in Plastic Biodegradation

Biodegradation of plastic is a process that results in total or partial conversion of organic carbon into biogas and biomass associated with the activity of a community of microorganisms (bacteria, fungi, and actinomycetes) capable of using plastic as a carbon source (Shah et al., 2008). Depending on the respiratory conditions (aerobic / anaerobic) and the microorganisms involved, the biogas will be different (CO_2_, CH_4_, H_2_S, NH_4_, and H_2_) (Mohee et al., 2008).

Microorganisms, including bacteria and fungi, present the capabilities to degrade or deteriorate plastics and several review papers updated the list of plastic-degraders (Shah et al., 2008; Bhardwaj et al., 2013; Kale et al., 2015; Pathak, 2017). *Arthrobacter*, *Corynebacterium*, *Micrococcus*, *Pseudomonas*, *Rhodococcus*, and *Streptomyces* were the prominent microbial taxa able to use plastic as sole carbon source and energy in laboratory conditions. Table 2 proposes an update of the current list of microorganisms proven to present biodegradation capabilities under laboratory conditions.

**Table 2:** List of microbial strains able to biodegrade various types of polymers.

Type of polymer	Strains	Reference
PE	*Brevibacillus borstelensis*	Hadad et al., 2005; Mohanrasu et al., 2018
	*Bacillus weihenstephanensis*	Ingavale and Raut, 2018
	*Comamonas* sp.	Peixoto et al., 2017
	*Delftia* sp.	Peixoto et al., 2017
	*Stenotrophomonas* sp.	Peixoto et al., 2017
	*Achromobacter xylosoxidans*	Kowalczyk et al., 2016
	*Bacillus* sp. YP1	Yang et al., 2014
	*Enterobacter asburiae* YT1	Yang et al., 2014
	*Bacillus amyloliquefaciens*	Das and Kumar, 2015
	*Bacillus pumilus* M27	Harshvardhan and Jha, 2013
	*Kocuria palustris* M16	Harshvardhan and Jha, 2013
	*Lysinibacillus xylanilyticus*	Esmaeili et al., 2013
	*Bacillus mycoides*	Ibiene et al., 2013
	*Bacillus subtilis*	Ibiene et al., 2013
	*Pseudomonas aeruginosa* PAO1 (ATCC 15729)	Kyaw et al., 2012
	*Pseudomonas aeruginosa* (ATCC 15692)	Kyaw et al., 2012
	*Pseudomonas putida* KT2440 (ATCC 47054)	Kyaw et al., 2012
	*Pseudomonas syringae* DC3000 (ATCC 10862)	Kyaw et al., 2012
	*Brevibacillus parabrevis*	Pramila, 2012
	*Acinetobacter baumannii*	Pramila, 2012
	*Pseudomonas citronellolis*	Pramila, 2012
	*Bacillus sphaericus*	Sudhakar et al., 2008
	*Rhodococcus ruber*	Gilan and Sivan, 2013
	*Aspergillus versicolor*	Pramila and Ramesh, 2011
	*Aspergillus* sp.	Pramila and Ramesh, 2011; Sheik et al., 2015
	*Chaetomium* sp.	Sowmya et al., 2012
	*Aspergillus flavus*	Sowmya et al., 2012
	*Penicillium simplicissimum*	Yamada et al., 2000; Sowmya et al., 2014
	*Lasiodiplodia theobromae*	Sheik et al., 2015
	*Paecilomyces lilacinus*	Sheik et al., 2015
	*P. pinophilum*, *A. niger*, *Gliocladium virens*, and *P. chrysosporium*	Manzur et al., 2004
	*Aspergillus glaucus* and *A. niger*	Kathiresan, 2003
PET	*Bacillus amyloliquefaciens*	Novotný et al., 2018
	*Nocardia* sp.	Sharon and Sharon, 2017
	*Ideonella sakaiensis*	Yoshida et al., 2016
	*Humicola insolens*	Ronkvist et al., 2009
	*Pseudomonas mendocina*	Ronkvist et al., 2009
	*Thermobifida fusca* (DSM 43793)	Müller et al., 2005
	*Penicillium citrinum*	Liebminger et al., 2007
	*Thermomonospora fusca*	Alisch et al., 2004
	*Fusarium oxysporum*	Nimchua et al., 2007
	*Fusarium solani*	Alisch et al., 2004; Nimchua et al., 2007
PHB	*Crupriavidus* sp.	Martínez-Tobón et al., 2018
	*Marinobacter algicola*	Martínez-Tobón et al., 2018
	Mixed cultures	Ansari and Fatma, 2016
	*Schlegella thermodepolymerans*	Romen et al., 2004
	*Caenibacterium thermophilum*	Romen et al., 2004
	*Acidovorax* sp. TP4	Kobayashi et al., 1999
	*Pseudomonas stutzeri*	Uefuji et al., 1997; Martínez-Tobón et al., 2018
	*Leptothrix discophora*	Takeda et al., 1998
	*Alcaligenes faecalis*	Tanio et al., 1982; Kita et al., 1995
	*Comamonas acidovorans* YM1609	Kasuya et al., 1997
	*Comamonas testosteroni*	Kasuya et al., 1997; Martínez-Tobón et al., 2018
	*Pseudomonas lemoignei*	Uefuji et al., 1997; Martínez-Tobón et al., 2018
	*Ralstonia pickettii*	Yamada et al., 1993; Martínez-Tobón et al., 2018
	*Pseudomonas fluorescens* YM1415 and nine Gram-	Mukai et al., 1994
	*Aspergillus niger*	Kumaravel et al., 2010
PHBV	*Clostridium botulinum*	Abou-Zeid et al., 2001
	*Clostridium acetobutylicum*	Abou-Zeid et al., 2001
	*Streptomyces* sp. SNG9	Mabrouk and Sabry, 2001
	*Pseudomonas lemoignei*	Jendrossek et al., 1993
	*Paecilomyces lilacinus*	Sang et al., 2001
PS	Strain TM1 and ZM1	Tang et al., 2017
	*Bacillus subtilis*	Asmita et al., 2015
	*Staphylococcus aureus*	Asmita et al., 2015
	*Streptococcus pyogenes*	Asmita et al., 2015
	*Exiguobacterium* sp.	Yang et al., 2015
	*Bacillus* sp NB6, *Pseudomonas aeruginosa* NB26, *Exiguobacterium* sp., *Microbacterium* sp. NA23, *Paenibacillus urinalis* NA26	Atiq et al., 2010
	*Rhodococcus ruber*	Mor and Sivan, 2008
	*Pseudomonas putida* CA-3 (NCIMB 41162)	Ward et al., 2006
	*Bacillus* sp. STR-Y-O	Oikawa et al., 2003
	Mixed microbial communities	Kaplan et al., 1979
	Mixed microbial communities (*Bacillus*, *Pseudomonas*, *Micrococcus*, and *Nocordia*)	Sielicki et al., 1978

*polyethylene (PE), polyethylene terephthalate (PET), polyhydroxybutyrate (PHB), and Poly(3-hydroxybutyrate-co-3-hydroxyvalerate) (PHBV), and polystyrene (PS). Detailed information on the origin of the strains and the methods used to prove biodegradation are available in the Supplementary Table S1.*

Biodegradation is considered to occur after or concomitant with physical and chemical degradation (abiotic degradation), which weakens the structure of polymers as revealed by roughness, cracks and molecular changes (İpekoglu et al., 2007). Alteration of plastic properties due to abiotic degradation is called “aging” and in nature depends on several factors such as temperature, solar light and chemicals that enhance the rate of degradation by oxidizing or disrupting the length of the polymer chain.

Biodegradation can be summarized in four essential steps, which have been described in detail in a review by Dussud and Ghiglione (2014):

- Bio-deterioration relates to the biofilm growing on the surface and inside the plastic, which increases the pore size and provokes cracks that weaken the physical properties of the plastic (physical deterioration) or releases acid compounds that modify the pH inside the pores and results in changes in the microstructure of the plastic matrix (chemical deterioration).
- Bio-fragmentation corresponds to the action of extracellular enzymes (oxygenases, lipases, esterases, depolymerases and other enzymes that may be as diverse as the large spectrum of polymer types) released by bacteria colonizing the polymer surface. These enzymes will reduce the molecular weight of polymers and release oligomers and then monomers that can be assimilated by cells.
- Assimilation allows oligomers of less than 600 Daltons to be integrated inside the cells to be used as a carbon source, thus increasing the microbial biomass.
- Mineralization is the ultimate step in the biodegradation of a plastic polymer and results in the excretion of completely oxidized metabolites (CO_2_, N_2_, CH_4_, and H_2_O).

### Rates of Plastic Degradation

Rates of degradation of conventional plastics by microorganisms are extremely low, even in optimized laboratory conditions (Krueger et al., 2015). Most of the conventional plastics are recalcitrant to biodegradation in marine and terrestrial environments, resulting in lifetimes of decades or even centuries (Krueger et al., 2015). Plastics present low bioavailability since they are generally solid and made of densely cross-linked polymers that provide low accessibility for microbes and enzymes circumscribed to the outermost layer of the items. In the pelagic ecosystem, plastics are biodegraded by the aerobic metabolism of microorganisms, i.e., the end product of the reaction will be microbial biomass, CO_2_ and H_2_O. The anaerobic biodegradation pathway would be more frequently encountered in sediment and is supposed to be even slower than in the pelagic zone (Ishigaki et al., 2004). Unfavorable C/N ratio is a key factor for biodegradation of other hydrocarbon-based products in the oceans (Sauret et al., 2016) and may potentially also limit plastic biodegration.

Data currently available rely heavily on culture-based approaches in laboratory conditions, although bacteria that can be cultured represent less than 1% of the number of bacteria in nature (the so-called “great plate count anomaly”) and a very small proportion of its very large diversity (Hugenholtz et al., 2009). To date, data on the rate of plastic mineralization in the oceans are still virtually non-existent. Congruent descriptions of the plastisphere that forms an abundant biofilm characterized by very diverse bacteria with active plastic-specific characteristics are available (Debroas et al., 2017; Dussud et al., 2018b). Evidence of pits visualized in the plastic debris that conform to bacterial shapes directly found in the marine environment (Zettler et al., 2013) together with a number of putative xenobiotic degradation genes likely involved in plastic degradation that were found to be significantly more abundant in the plastic-specific communities (Bryant et al., 2016; Debroas et al., 2017; Dussud et al., 2018b) are thus of great interest. A recent study underlined the need of cometabolic pathways on PE biodegradation, thus confirming that complex microbial communities rather than single species are necessary to degrade recalcitrant plastic (Syranidou et al., 2017). So far, the timescales of degradation and the characterization and the fate of the degradation products, are fundamental, yet still unanswered questions.

### Standard Tests for Plastic Biodegradability at Sea

**FIGURE 2:**
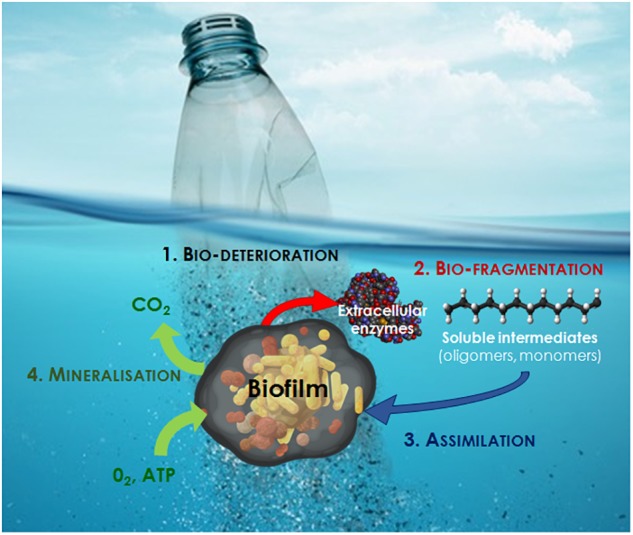
The different steps of plastic biodegradation at sea (modified from Dussud and Ghiglione, 2014).

**FIGURE 3:**
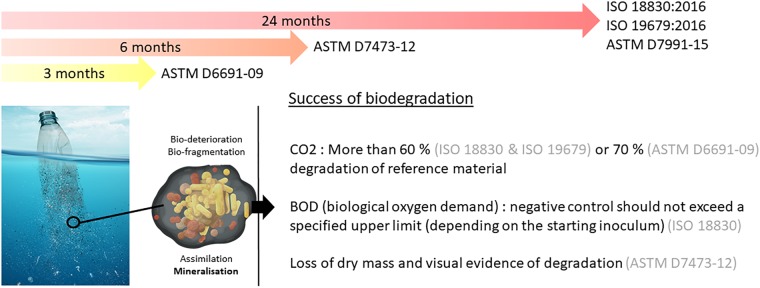
Current standards on biodegradability of plastics at sea. ISO 18830: Plastics-determination of aerobic biodegradation of non-floating plastic materials in a seawater/ sandy sediment interface – Method by measuring the oxygen demand in closed respirometer. ISO 19679: Determination of aerobic biodegradation of non-floating plastic materials in a seawater/sediment interface – method by analysis of evolved carbon dioxide respirometer. ASTMD7991: Determining aerobic biodegradation of plastics buried in sandy marine sediment under controlled laboratory conditions. ASTM D7473: Standard test method for weight attrition of plastic materials in the marine environment by open system aquarium incubations. ASTM: Standard test method for determining aerobic biodegradation of plastic materials in the marine environment by a defined microbial consortium or natural sea water inoculum.

The current standards for marine environments propose tests based on respirometry measurements, susceptible to describe the mineralization step of plastic biodegradation in aerobic conditions (see Figure 2). They impose a minimum percentage of conversion from plastic to CO_2_ ranging from 60 to 70% over a period of 3 months (ASTM D6691-09), 6 months (ASTM D7473-12), or 24 months (ISO 18830, ISO 19679, ASTM D7991-15) under aerobic conditions (see Figure 3). Anaerobic biodegradation is characterized by specific standards (see for example ASTM D5511-18), but to our knowledge none of these standards applies to the marine environment. Biodegradation of a plastic is characterized by the time required to achieve mineralization under controlled conditions. These tests cannot be considered as a proof of ready biodegradability (total conversion of plastic into biomass and CO_2_), but rather an indication about a potential for biodegradation in the oceans.

Recently, these standards were considered insufficient in their ability to realistically predict the biodegradability in marine environment (Harrison et al., 2018). These tests can significantly underestimate the time required for polymer biodegradation within natural ecosystems. First, the authors underlined “biases associated with the preparation of experimental inocula and the test conditions themselves, including the use of preselected and/or pre-conditioned strains, artificially modified inocula, powdered test materials, nutrient-rich synthetic media and test temperatures that are frequently higher than those encountered within the environment.” The authors also pointed out “the lack of clear guidelines for the analysis of different polymer types, including composite materials and plastics that contain additives,” which can considerably influence the rates of biodegradation. “There is also a paucity of guidelines for materials of varying shapes and sizes and, in certain cases, the test procedures lack a sufficient level of statistical replication.” Another concern, not raised by Harrison et al. (2018), is the biases associated with the common method for determining biodegradability, i.e., measurements of CO_2_ evolution. This method may lead to either underestimation or overestimation of the plastic biodegradation due to other processes. It is noteworthy that plastic generally presents high sorption capability of organic matter (especially hydrophobic organic chemicals including pollutants) that can be biodegraded by the plastisphere biofilm, thus resulting in a CO_2_ production that has nothing to do with plastic biodegradation (Lee et al., 2014). Inversely, several papers reported the importance of photosynthetic microorganisms growing on plastics, which consume CO_2_ regardless of plastic biodegradation (Zettler et al., 2013; Bryant et al., 2016; Dussud et al., 2018b). Further studies are needed to evaluate the relative degree of CO_2_ consumption by photosynthesis, CO_2_ production by organic matter degradation by the plastisphere as compared to CO_2_ production due to plastic biodegradation.

The limitations of the respiratory methods described above can be overcome by other additional analytical techniques and approaches to confirm changes in the physical properties and the chemical structure of polymers during biodegradation. Alterations in the visual appearance and in the mass or changes in mechanical properties are relatively easy and low-cost methods for the evaluation of physical changes during biodegradation. Other methods could be combined to confirm changes in the molecular structure of polymers, such as measurements of surface hydrolysis and other chromatographic (gas chromatography with or without flame ionization detection, liquid chromatography, gel-permeation chromatography) measurements coupled or not with spectrometric techniques (mass spectrometry, nuclear magnetic resonance spectroscopy, Fourier-transform infrared spectroscopy). Optical, atomic force and scanning electron microscopy can also be used to assess the biodeterioration of the surface due to microbial activity or biofilm formation. Any of these techniques are enough to prove biodegradation by its own, and each of them has limitations that have been previously detailed for example in the excellent reviews of (Koutny et al., 2006; Harrison et al., 2018; Ho et al., 2018). The current standards sometimes propose to use such techniques to corroborate the main test based on respirometry measurement, but no clear guidelines on how to use these tests is provided.

### Examples of Metabolic Pathways of Polymer Biodegradation

There are currently more than 5,300 grades of synthetic polymers for plastics in commerce (Wagner and Lambert, 2018). They are generally produced with a range of chemical additives such as plasticizers, flame retardants, antioxidants and other stabilizers, pro-oxidants, surfactants, inorganic fillers or pigments (Wagner and Lambert, 2018). Their heterogeneous physical-chemical properties will likely result in very heterogeneous metabolic pathways of biodegradation, especially when considering the large variety of microorganisms that may interact for the degradation of a single piece of plastic, together with the environmental factors of very dynamic oceanic conditions. We are aware that treating plastic as a single compound does not make sense and providing details on the metabolic pathways of plastic biodegradation would necessarily be unrepresentative of the complexity of the various processes that occur in the environment. We have chosen to focus on the metabolic pathways associated with the biodegradation of model compounds used in the formulation of conventional (PE, PET, and PS) and so called “biodegradable” plastics (PHA) that are the most popular and the most extensively studied in the literature. Moreover, it should be noted that because of the difficulty of dealing with long-term experiments and complex communities under natural conditions, all the following studies describing the metabolic pathways of plastic biodegradation were done using a culture-based approach.

#### Metabolic Pathways of Polyethylene (PE) Biodegradation

**FIGURE 4:**
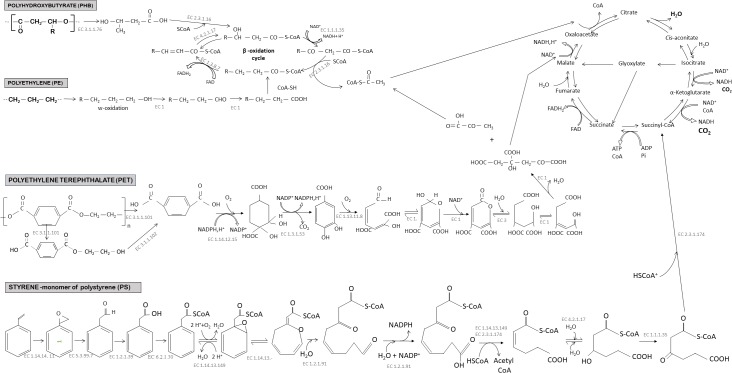
Biodegradation pathways under aerobic conditions of three conventional plastics (polyethylene, polyethylene terephthalate, and polystyrene) and one biodegradable plastic (polyhydroxybutyrate). See explanation in the text indicating that degradation rates may be very different between polymer types. Complete mineralisation into CO_2_ and H_2_O occurred after several steps of transformation of the initial molecule involving several microbial enzymes. The common stage of transformation through the TCA cycle produce also ATP, which is a key component for bacterial growth and biomass production. Enzyme commission numbers (EC numbers) were given for each enzyme-catalyzed reactions. EC 3.1.1.76, poly(3-hydroxyoctanoate) depolymerase; EC 2.3.1.16, acetyl-CoA C-acyltransferase; EC 1.1.1.35, 3-hydroxyacyl-CoA dehydrogenase; EC 1.3.8.7, medium-chain acyl-CoA dehydrogenase; EC 4.2.1.17, enoyl-CoA hydratase; EC 3.1.1.101, poly(ethylene terephthalate) hydrolase; EC 3.1.1.102, Mono(2-hydroxyethyl) terephthalate hydrolase; EC 1.14.12.15, terephthalate 1,2-dioxygenase; EC 1.3.1.53, 3,4-dihydroxycyclohexa-1,5-diene-1,4-dicarboxylate dehydrogenase; EC 1.13.11.8, protocatechuate 4,5-dioxygenase; EC 1, Oxidoreductase; EC 3, Hydrolase; EC 1.14.14, 11 styrene monooxygenase; EC 5.3.99.7, styrene-oxide isomerase; EC 1.2.1.39, phenylacetaldehyde dehydrogenase; EC 6.2.1.30, phenylacetylCoA ligase; EC 1.14.13.149, phenylacetyl-CoA 1,2-epoxidase; EC 1.14.13, ring 1,2-epoxyphenylacetyl-CoA isomerase; EC 1.2.1.91, 3-oxo-5,6-dehydrosuberyl-CoA semialdehyde dehydrogenase; EC 2.3.1.174, 3-oxoadipyl-CoA/3-oxo-5,6-dehydrosuberyl-CoA thiolase; EC 4.2.1.17, 2,3-dehydroadipyl-CoA hydratase; EC 1.1.1.35, 3-hydroxyadipyl-CoA dehydrogenase.

High- and low-density polyethylene is a long linear carbon chain (CH_2_) belonging to the family of polyolefins. Polyethylene is derived from petroleum sources and its large use in our daily life made it the first plastic waste found at sea surface. PE is considered difficult to biodegrade because the long chains of carbons and hydrogens are very stable and contain very balanced charges. Microorganisms generally need imbalance of electric charge to perform biodegradation. To destabilize the local electric charge, bacteria use oxygenases: enzymes able to add oxygen to a long carbon chain (Krueger et al., 2015). For instance, mono-oxygenases and di-oxygenases incorporate, respectively, one and two oxygen atoms, forming alcohol or peroxyl groups that are less recalcitrant for biodegradation. Oxidation may also be processed by abiotic reactions associated with UV radiation or temperature (for more details, see the review by Singh and Sharma, 2008). Oxidation of PE results in the formation of carboxylic groups, alcohols, ketones, and aldehydes by a radical reaction (Vasile, 1993; Gewert et al., 2015). The oxidation and fragmentation of PE make the polymer more hydrophilic and facilitates access to other extracellular enzymes, such as lipases and esterases after the formation of carboxylic groups, or endopeptidases for amide groups (Gewert et al., 2015). Other enzymes such as laccase in *Rhodococcus ruber* are excreted and can facilitate the biodegradation of PE (Santo et al., 2013). Interestingly, a recent study focused on soluble oxidized oligomers showed that 95% of these compounds were assimilated by a strain of *Rhodococcus rhodochrous* after 240 days of incubation (Eyheraguibel et al., 2017). The polymer is broken down into small oligomers of 600 Da incorporated in the cells by carriers belonging to the Major Facilitor Superfamily (MFS) or harboring ATP binding cassettes (ABC) (Gravouil et al., 2017). β-oxidation transforms oxidized carboxylic molecules (having an even number of carbon atoms) into acetyl coA or propionyl coA (if odd number of carbons). Carboxylation of propionyl coA into succinyl coA is performed by propionyl-coA carboxylase. Gravouil et al. (2017), propose identification of an overexpressed enzyme, when the bacteria find PE in the medium (Gravouil et al., 2017). Acetyl coA and succinyl coA enter the tricarboxylic acid (TCA) cycle (Figure 4). This cycle produces chemical energy in the form of a reducing power (NADH, H + and CoQ_10_H_2_) used in the respiratory chain to produce ATP, which is necessary to create new microbial biomass *via* replication processes. It also produces CO_2_ and H_2_O that sign the complete mineralisation of PE.

For 20 years now, scientists have been interested in the biodegradation of polyethylene by the microbial community. Bacterial and fungal strains presenting biodegradation capabilities of PE are listed in Table 2 and Supplementary Table S1.

Genetic evidence of PE biodegradation remains scarce in the literature, but preliminary work highlighted enzymes, transporters or genes that may be involved in this process (Gravouil et al., 2017). Alkane hydroxylase genes were found to play a central role in PE biodegradation for *Pseudomonas* sp. E4 strain, which was capable of mineralizing 28.6% of the organic carbon of the polymer in 80 days. The alkB gene was then introduced in *Escherichia coli* BL21 strain, which was then able to mineralize 19.3% of the organic carbon of the polymer (Yoon et al., 2012). Only two other studies used genetic analysis to provide evidence for the importance of laccase in PE biodegradation by *R. ruber* (Sivan, 2011; Santo et al., 2013; Gravouil et al., 2017).

#### Metabolic Pathways of Polyethylene Terephthalate (PET) Biodegradation

Polyethylene terephthalate is part of the polyester family and it is widely used in the design of bottles and synthetic fibers. It is considered as persistent plastic in the environment because of its long carbon chains containing aromatic rings that are difficult to biodegrade (Marten et al., 2005). In recent years, studies have shown that some bacterial strains were able to degrade PET as sole carbon source and energy, such as *Ideonella sakaiensis* (Yoshida et al., 2016), *Nocardia* sp. (Sharon and Sharon, 2017) *Pseudomonas mendocina* (Ronkvist et al., 2009), *Thermobifida fusca* (Müller et al., 2005). Some fungal communities are also known to biodegrade PET, such as *Humicola insolens*, several *Fusarium* species, and *Penicillium citrinum* (Silva et al., 2005; Liebminger et al., 2007; Nimchua et al., 2007; Ronkvist et al., 2009). Cutinases or hydrolases play key roles in PET biodegradation (Danso et al., 2018). For example, *I. sakaiensis* 201-F6 adhered to the PET surface and first secreted two enzymes involved in the biodegradation process of PET: PETase (hydrolase) and MHETase. PETase is an extracellular enzyme capable of hydrolysing PET to mono-(2-hydroxyethyl) terephthalate (MHET), terephthalic acid (TPA), and bis (2-hydroxyethyl) terephthalate (BHET). Fungi seem to have the same biodegradation strategy and are able to degrade PET into BHET and MHET (Liebminger et al., 2007). The MHETase hydrolyzes MHET to TPA and ethylene glycol (EG). The terephthalic acid molecule is then internalized in the bacterial cells by the TPA transporter (Hosaka et al., 2013) and then catabolized by TPA 1,2-dioxygenase (TPADO) and 1,2-dihydroxy-3,5-cyclohexadiene-1,4-dicarboxylate dehydrogenase (DCDDH) to give protocatechuic acid (PCA) as the final molecule (Yoshida et al., 2016). This PCA is cleaved by PCA 3,4 dioxygenase (PCA34) to give the hemiacetal form of 4-carboxy-2-hydroxymuconic. The latter becomes the substrate of a dehydrogenase to form 2-pyrone-4,6-dicarboxylic acid that enters the TCA cycle and initially transformed into pyruvate and oxaloacetate, then assimilated as CO_2_ and H_2_O (Figure 4).

#### Metabolic Pathways of Polystyrene (PS) Biodegradation

Polystyrene is a polymer composed of styrene monomers (CH_2_ = CH_2_-Ph). The polymer is highly hydrophobic and presents a high molecular weight. Like other conventional plastics, partial biodegradation in the laboratory has been observed while it continues to accumulate in the oceans (Auta et al., 2017) thus inciting increasing interest in PS biodegradation (see Supplementary Table S1; Oikawa et al., 2003; Mor and Sivan, 2008; Atiq et al., 2010; Asmita et al., 2015; Yang et al., 2015; Tang et al., 2017).

Several biodegradation pathways may be considered, depending on the microorganism involved. The predominant pathway is the oxidation pathway of the styrene side chain presented in Figure 4. The styrene is directly oxidized with a styrene monooxygenase to form a styrene epoxide which will then be oxidized to phenylacetaldehyde by styrene oxide. This molecule is then catabolized into phenylacetic acid. This conversion of styrene to phenylacetic acid is called the upper pathway of styrene metabolism. Phenylacetic acid is converted to phenylacetyl-CoA (acetyl coenzyme A) by the so-called lower pathway (Luu et al., 2013) then subjected to several enzymatic reactions (Figure 4) to finally enter the tricarboxylic acid (TCA) cycle. The biodegradation products enter the TCA cycle through the final formation of acetyl-Co A and succinyl-CoA (succinyl-CoenzymeA) (Luu et al., 2013).

Interestingly, *Pseudomonas putida* CA-3 can accumulate polyhydroxyalkanoates (PHA at medium chain length) when growing on styrene, thus using an original biodegradation pathway. A catabolic operon has been identified as responsible for this bioconversion; this path is called the PACoA (Phenylacetyl-CoA) catabolon. It involves oxidation of the aromatic ring, followed by entry into the β-oxidation cycle and the conversion to acetyl-CoA (O’Leary et al., 2005). This acetyl-CoA can follow different metabolic pathways, either entering the TCA cycle or following the *de novo* fatty acid biosynthesis path which will give as final product medium-chain-length polyhydroxyalkanoates (mcl-PHAs) (O’Leary et al., 2005). This study shows the complexity of studying the biodegradation pathways of these polymers and indicates the great range of possibilities when considering the large diversity of microorganisms found in the plastisphere.

#### Metabolic Pathways of Polyhydroxyalkanoate (PHA) Biodegradation

The current global production of PHA is increasing, reaching 49,200 tons per year that represents 2.4% of the production of bioplastics^1^. PHAs are biopolymers of hydroxylated fatty acids produced within a bacteria in granular form. Each PHA monomer ([CO-CH_2_-CHR-O]_n_) consists of hydroxyalkanoates linked together by ester bonds. The alkyl group (R) varies from a methyl group to a tetradecyl group. When bacteria are placed in a medium with an excess carbon source and low nutrient content, they accumulate storage granules. Over 300 bacterial species are capable of producing 80 different hydroxyalkanoate monomers, and some bacteria can accumulate up to 90% of their total weight of polymer in very specific conditions (Peña et al., 2014). One of the most commonly used PHA for plastic production is polyhydroxybutyrate (PHB), which has a methyl as an alkyl group (R) ([CO-CH2-CHCH3-O]_n_). PHB is one of the homopolymers with high commercial power because it has thermoplastic, hydrophobic, low oxygen permeability and is considered biodegradable (Mothes et al., 2004; Chang et al., 2012). It is not very deformable, because of its high crystallinity (Gorke et al., 2007) and it has a high melting point close to its thermal degradation temperature (Reis et al., 2003). A copolymer made of Poly(3-hydroxybutyrate-co-3-hydroxyvalerate) (PHBV) that reduces the melting point of PHB is seen to emerge in PHA production. The advantage of using PHA is that it is stable over time, as long as the conditions governing its biodegradation are not met (Jaffredo et al., 2013).

Due to their microbial origin, PHAs were found to be biodegradable in many environments such as soil, marine ecosystems or sewage sludge (Eubeler et al., 2010). Biodegradation of Poly(3-hydroxybutyrate-co-3-hydroxyhexanoate) has been proven with comparable rates to that of cellulose, with faster degradation found under aerobic (85 days) compared to anaerobic (6 months) conditions (Wang et al., 2018). The biodegradation scheme in Figure 4 shows the different steps of PHB biodegradation. When the biodegradation is not carried out inside the cells by bacteria that produce their own PHB, other bacteria initiate the biodegradation of PHB in the medium by external hydrolysis using ectoenzymes that convert the polymers into hydroxylated acid monomers of hydroxybutyrate (HB) (Peña et al., 2014). This molecule is water soluble and small enough to passively diffuse across the bacterial membrane and enter the β-oxidation cycle. The resulting acetyl-CoA will be oxidized in the TCA cycle until final mineralisation (Alshehrei, 2017). PHA-degradation has been proven in the laboratory under aerobic or anaerobic conditions (see a non-exhaustive list in Supplementary Table S1). The dominant bacteria in aerobic marine conditions belong to *Clostriales*, *Gemmatales*, *Phycisphaerales*, and *Chlamydiales*, whereas *Cloacamonales* and *Thermotogales* dominate in anaerobic sludge (Wang et al., 2018).

## Concluding Remarks

In this review, we have presented both aspects of microbial ecotoxicology on marine plastic debris, namely the impact of plastic on marine microbial life and inversely how microbes can play a role in plastic biodegradation. An increasing number of studies either describe the different steps of biofilm formation under marine conditions, or give new insights on bacteria colonizing the aged plastics directly sampled at sea. The very diverse and active bacteria living on plastics as compared to the surrounding waters suggest a potential impact on the global biogeochemical cycles associated with the relatively recent introduction of plastic in the oceans, impact that remains to be determined. Plastic released in the oceans is also accused to be a raft for invasive species including pathogenic bacteria, but no proof of pathogenicity on marine animals or humans in relation to plastic ingestion has emerged so far.

A better knowledge of the plastisphere is also a critical issue in understanding the role played by bacteria in plastic biodegradation. Several studies have underlined that current standards are failing to prove biodegradability at sea for several reasons that have been highlighted in this review. Biodegradation of a polymer at sea depends on many factors related to its own composition, but also on the various ecosystems and environmental conditions encountered during its very long lifetime. It is for these reasons that plastic polymers continue to accumulate at sea and that biodegradation rates reported in the laboratory are never reached in the environment. Thus, a complete study of the biodegradation of a polymer at sea must combine several monitoring parameters, and especially be confirmed in the field with experiments *in situ*. Given the complexity of the plastic problem, research network initiatives such as “Polymers & Oceans” that bring together physicists, chemists and biologists are required to answer the wishes and needs of many scientists to face this environmental problem and its resonance in the society.^2^

## Author Contributions

J-FG designed the general plan of the review. JJ, A-LM, JC, and J-FG made the figures. JJ, JC, CO, CP, PC, MP-P, VB, A-LM, and J-FG wrote the manuscript and approved the final version.

## Conflict of Interest Statement

The authors declare that the research was conducted in the absence of any commercial or financial relationships that could be construed as a potential conflict of interest.

## Footnotes

1 https://www.european-bioplastics.org

2 https://po2018.wixsite.com/po2018

## Supplementary Material

TABLE S1 Detailed information on the origin and methodology used to prove the biodegradation of various polymer types (PE, PET, PHB, PHBV, and PS) by microorganisms cited in Table 1.
