# Supplementary material for: Microbial Ecotoxicology of Marine Plastic Debris: A Review on Colonization and Biodegradation by the “Plastisphere”
Source: Front Microbiol. 2019 Apr 25;10:865. doi: 10.3389/fmicb.2019.00865 (PMC6497127; doi:10.3389/fmicb.2019.00865)
Supplement: TABLE S1 — Detailed information on the origin and methodology used to prove the biodegradation of various polymer types (PE, PET, PHB, PHBV, and PS) by microorganisms cited in Table 1. [file Table_1.docx]

| Type of polymer | Strains | Origin | Proof of degradation | Reference |
| --- | --- | --- | --- | --- |
| Polyethylene  (PE) | *Brevibacillus borstelensis* | Soil & Sea water | Decrease of gravimetry of 30 % and molecular weight of 11 % | Hadad et al., 2005; Mohanrasu et al., 2018 |
|  | *Bacillus Weihenstephanensis* | Soil | 7% weight loss in 6 month | Ingavale and Raut, 2018 |
|  | *Comamonas* sp. | Soil | Decrease of crystallinity due to assimilation of the lower molecular weight fragments. Metabolic activity and cellular viability after 90 days of incubation with PE as the sole carbon source. Chemical modification: chain scission, formation of alkoxy groups, acyl groups, nitro groups, double bonds and carbonyl groups | Peixoto et al., 2017 |
|  | *Delftia* sp. | Soil | Decrease crystallinity of PE. Metabolic activity and cellular viability after 90 days of incubation with PE as the sole carbon source. Chemical modification: chain scission, formation of alkoxy groups, acyl groups, nitro groups, double bonds and carbonyl groups | Peixoto et al., 2017 |
|  | *Stenotrophomonas* sp. | Soil | Increase crystallinity of PE, potential to break the long alkane chain. Metabolic activity and cellular viability after 90 days of incubation with PE as the sole carbon source. Chemical modification: chain scission, formation of alkoxy groups, acyl groups, nitro groups, double bonds and carbonyl groups | Peixoto et al., 2017 |
|  | *Achromobacter xylosoxidans* | Soil | 9 %weight loss in 150 days, oxidization of PE, embodying an oxygen atom in the damaged structure of the PE chain. Damages of the HDPE film surface | Kowalczyk et al., 2016 |
|  | *Bacillus sp YP1* | Guts of waxworm | 10.7 % weight loss in 28 days, decrease of hydrophobicity, molecular weight shift | Yang et al., 2014 |
|  | *Entobacter asburiae YT1* | Guts of waxworm | 6.1 % weight loss in 28 days, decrease of hydrophobicity and molecular weight shift | Yang et al., 2014 |
|  | *Bacillus amyloliquefaciens* | Soil | 11-16 % weight loss and 12-14.7 % mineralization level in 60 days | Das and Kumar, 2015 |
|  | *Bacillus pumilus M27* | Sea water | Viability and activity after incubation with PE as the sole carbon source. 1.5% weight loss in 30 days. | Harshvardhan and Jha, 2013 |
|  | *Kocuria palustris M16* | Sea water | Viability and activity after incubation with PE as the sole carbon source. 1 % weight loss in 30 days. | Harshvardhan and Jha, 2013 |
|  | *Lysinibacillus xylanilyticus* | Soil | Production of 734 mg of CO_2_ after 126 days, use of carbonyl groups of irradiated PE. Decrease of the carbonyl index (less band between 1000 and 1700 cm-1). | Esmaeili et al., 2013 |
|  | *Bacillus mycoides* | Mangrove swamp | 8.41% weight loss in 2 months, decrease carbonyl index between 10,5-13.7 % | Ibiene et al., 2013 |
|  | *Bacillus subtilis* | Mangrove swamp | 23.15 % weight loss in 2 months, decrease carbonyl between 10.5-13.7 % | Ibiene et al., 2013 |
|  | *Pseudomonas aeruginosa PAO1 (ATCC 15729)* | Laboratory strain | 20 % weight loss in 120 days, decrease of tensile strength and 80% carbonyl index, surface changes (micro cracks, deformation) | Kyaw et al., 2012 |
|  | *Pseudomonas aeruginosa (ATCC 15692)* | Laboratory strain | 11 % weight loss in 120 days, decrease of tensile strength and 16 % carbonyl index, surface changes (micro cracks, deformation) | Kyaw et al., 2012 |
|  | *Pseudomonas putida (KT2440 ATCC 47054)* | Laboratory strain | 11 % weight loss in 120 days, decrease of tensile strength and 28 % carbonyl index, surface changes (micro cracks, deformation) | Kyaw et al., 2012 |
|  | *Pseudomonas syringae (DC3000 ATCC 10862)* | Laboratory strain | 11.3 % weight loss in 120 days, decrease of tensile strength and 78 % carbonyl index, surface changes (micro cracks, deformation) | Kyaw et al., 2012 |
|  | *Brevibacillus parabrevis* | Soil | Production of 0.7042 g/L in Sturm test during 48h | Pramila, 2012 |
|  | *Acinetobacter baumannii* | Soil | Production of 1.0603 g/L in Sturm test during 48h | Pramila, 2012 |
|  | *Pseudomonas citronellolis* | Soil | Production of 0.5706 g/L in Sturm test during 48h | Pramila, 2012 |
|  | *Bacillus sphaericus* | Sea water | 3.5-10% weight loss. Decrease in crystallinity by 8% for LDPE and by 2.2% for HDPE. The surface becomes more hydrophilic. Decrease of the carbonyl index | Sudhakar et al., 2008 |
|  | *Rhodococcus rubber* | Soil | 30 days of incubation degraded 8% gravimetrically, carbonyl index reduction of 66% | Gilan and Sivan, 2013 |
|  | *Aspergillus versicolor* | Sea water | Increase of fresh weight and CO_2_ production of 3.8 g/L in Sturm test in one week. Appearance of cracks and holes at the surface | Pramila et al.,2011 |
|  | *Aspergillus sp.* | Sea water | Increased fresh weight and CO_2_ production of 4 g/L in Sturm test in one week. Appearance of cracks and holes at the surface | Pramila et al.,2011 |
|  | *P. pinophilum, A niger, Gliocladium virens* & *P. chrysosporium* | Laboratory strain | Apparition of product of degradation (carbonyl & double bonds groups), decreases melting temperature, melting point andmean crystallite size | Manzur et al., 2004 |
|  | *Aspergillus glaucus* et *A. niger* | Mangrove | 20.54 % weight loss in 1 month | Kathiresan et al., 2003 |
| Polyethylene terephthalate  (PET) | *Bacillus amyloliquefaciens* | Compost | Decrease of index carbonyl and increase of molecular weight with disappeared low molar weight LLDPE oligomers | Novotný et al., 2018 |
|  | *Nocardia sp.* | Soil | Chemical modification of polymeric chains, impact on the crystalline structure of PET. Weight loss of 8% and decrease in tensile strength (loss of 6 Pa) after 200 days. | Sharon and Sharon, 2017 |
|  | *ideonella sakaiensis* | Activated sludge | Degraded PET film surface (0,13 mg.cm^-2^.day^-1^) | Yoshida et al., 2016 |
|  | *Humilicas insolens* | Laboratory strain | Assay of cutinase activity that hydrolyzes PET, measuring NaOH consumption, 97% weight loss | Ronkvist et al., 2009 |
|  | *Pseudomonas mendocina* | Laboratory strain | Detection of product of biodégradation (TPA) after 96 h. Weight loss of 5 % in 96 h. Contain enzyme able to transform TPA in ethylene glycol | Ronkvist et al., 2009 |
|  | *Thermobifida fusca DSM 43793* | Laboratory strain | 50 % weight loss in 3 weeks. Decrease in the film thickness between 8- and 17-mm. Isolation and identification of hydrolases. | Müller et al., 2005 |
| Polyhydroxy butyrate  (PHB) | *Crupriavidus* sp. | Activated sludge | 90 % weight loss in 1 week. Increase in protein concentration (initial concentration at 50 μg/mL and after 150 h: 92 μg/mL) | Martínez-Tobón et al., 2018 |
|  | *Marinobacter algicola* | Laboratory strain | 5% weight loss in 1 week. No present a halo of degradation (clear zone). | Martínez-Tobón et al., 2018 |
|  | Mix cultures | Soil | 24.58 % weight loss in 60 days | Ansari and Fatma, 2016 |
|  | *Schlegella thermodepolymerans* | Compost | Degradation halo (clear zone) on agar plate with PHB after 2 days | Romen et al., 2004 |
|  | *Caenibacterium thermophilum* | Compost | Degradation halo (clear zone) on agar plate with PHB after 2 days | Romen et al., 2004 |
|  | *Acidovorax* Sp. Strain TP4 | environmental sample | Extracellular PHB depolymerase expressed decreasing the turbidity of a PHB suspension. | Kobayashi et al., 1999 |
|  | *Pseudomonas stutzeri* | Sea water | Use P(3HB) as carbon source. 15 % weight loss after 24 h. Characterisation and isolation of PHB depolymerase | Martínez-Tobón et al., 2018; Uefuji et al., 1997 |
|  | *Leptothrix discophora* | Hot spring | Halo of degradation (clear zone) on agar plate due to degradation of PHB | Takeda et al., 1998 |
|  | *Alcaligenes faecalis* | Sea water and activated sludge | Use PHB as a sole source of carbon. Identification and characterisation of PHB depolymerase | Kita et al., 1995; Tanio et al., 1982 |
|  | *Comamonas acidovoransa* YM1609 | Lake | Use PHB and PHBV in liquid media as only source carbon. | Kasuya et al., 1997 |
|  | *Comamonas tetsteroni* | Soil | on source PHB as carbon source. 53 % weight loss in 1 week. Characterisation and isolation of PHB depolymerase | Kasuya et al., 1997; Martínez-Tobón et al., 2018 |
|  | *Pseudomonas lemoignei* | Laboratory strain | Use PHB as carbon source. Halo of degradation on petri dish (clear zone). 15 % weight loss in 150 h | Martínez-Tobón et al., 2018; Uefuji et al., 1997 |
|  | *Ralstonia pickettii* | Atmosphere in Japan | Use PHB as carbon source. Halo of degradation on Petri dish (clear zone) in 7 days. Increase of protein concentration to 89 μg/mL | Martínez-Tobón et al., 2018; Yamada et al., 1993 |
|  | *Pseudomonas fluorescens* (YM1415) and 9 Gram- | Lake | Halo of degradation (clear zone) on agar plate | Mukai et al., 1994 |
| PHBV  (Polyhydroxy butyrate and valerate) | *Clostridium botulinum* | Laboratory strain | Halo of degradation (clear zone) on agar plate | Abou-Zeid et al., 2001 |
|  | *Clostridium acetobutylicum* | Waste water sludge | Halo of degradation (clear zone) on agar plate | Abou-Zeid et al., 2001 |
|  | *Streptomyces* sp. SNG9 | Sea water | Formation of clear zone and alteration of polymers sheets | Mabrouk and Sabry, 2001 |
|  | *Pseudomonas lemoignei* | Laboratory strain | Utilization of carbon sources for growth, detection of translucent halos appearing in the opaque top agar layer | Jendrossek et al., 1993 |
| Polystyrene  (PS) | Strain TM1 and ZM1 | Guts of *Tenebrio molito*r and *Zophobas moria* | Use PS as a carbon source. Decreased turbidity in a liquid culture medium with PS as a carbon source. | Tang et al., 2017 |
|  | *Bacillus subtilis* | Soil | 20-58.8% weight loss in 4 months | Asmita et al., 2015 |
|  | *Staphylococcus aureus* | Soil | 4-37% weight loss depending on the medium used after 4 months | Asmita et al., 2015 |
|  | *Streptococcus pyogenes* | Soil | 8.33-11.11 % weight loss depending on the medium used after 4 months | Asmita et al., 2015 |
|  | *Exiguobacterium* sp. | Mealworms | 7.4 % of weight loss in 60 days | Yang et al., 2015 |
|  | *Bacillus* sp NB6, *Pseudomonas aeruginosa* NB26, *Exiguobacterium* sp., *Microbacterium* sp. NA23, *Paenibacillus urinalis* NA26 | Soil | Production of intermediate products of biodegradation in 8 weeks. No weight loss or change in surface | Atiq et al., 2010 |
|  | *Rhodococcus ruber* | Soil | 0.8 % of gravimetric weight loss in 8 weeks | Mor and Sivan, 2008 |
|  | *Pseudomonas putida* CA-3 (NCIMB 41162) | Laboratory strain | Pyrolysis of PS to styrene oil and the final product is PHA. 1 g of styrene oil was converted to 0.0625 g of PHA and 0.025 bacterial biomass | Ward et al., 2006 |
|  | *Bacillus* sp. STR-Y-O |  | 40 % weight loss for styrene and 56 % for polystyrene in 8 days | Oikawa et al., 2003 |
|  | Mixed microbial communities | Soil | 0.04 %-0.57 % of CO_2_ production between 5 - 11 weeks | Kaplan et al., 1979 |
|  | Mixed microbial communities (*Bacillus, Pseudomonas, Micrococcus* and *Nocordia*) | Soil | 1.5 - 3% of biodegradation by respirometry method during 4 months | Sielicki et al., 1978 |
